# Placa horizontal em banda de tensão: Uma nova estratégia para fraturas desviadas e multifragmentares da tuberosidade anterior da tíbia associadas a fraturas metafisárias complexas

**DOI:** 10.1055/s-0046-1822833

**Published:** 2026-07-14

**Authors:** Robinson Esteves Pires, Gustavo Waldolato Silva, Rafael Chagas Silva, Erick Veiga Franco da Rosa

**Affiliations:** 1 Serviço de Ortopedia e Traumatologia. Hospital Felício Rocho, Belo Horizonte, MG, Brasil; 2 Departamento do Aparelho Locomotor, Faculdade de Medicina, Universidade Federal de Minas Gerais, Belo Horizonte, MG, Brasil

**Keywords:** fraturas do joelho, fraturas do planalto tibial, tíbia, knee fractures, tibia, tibial plateau fractures

## Abstract

Não existe padronização de tratamento para as fraturas multifragmentares da tuberosidade anterior da tíbia (TAT) nos pacientes adultos, com ou sem associação com fraturas metafisárias da tíbia proximal. O objetivo desta nota técnica é demonstrar os resultados da utilização da placa de minifragmentos colocada segundo o princípio da banda de tensão, de forma horizontal, em dois pacientes portadores de fratura multifragmentar da TAT.

## Abstract (en)

There is no standardized treatment for multifragmentary fractures of the anterior tibial tuberosity (ATT) in adult patients, with or without concomitant metaphyseal fractures of the proximal tibia. This technical note aims to demonstrate the results of using a minifragment plate positioned horizontally according to the tension band principle in two patients with multifragmentary fractures of ATT.

## Introdução

As fraturas de alta energia da região proximal da tíbia que envolvem a tuberosidade anterior (TAT) representam um desafio significativo no âmbito ortopédico devido à sua complexidade biomecânica. Essas lesões são frequentemente causadas por traumas de alta energia cinética, resultando em fraturas multifragmentares e associadas a acometimentos extensos das partes moles circundantes, o que dificulta ainda mais o manejo clínico e cirúrgico. 1 2 3 A TAT é o ponto de inserção do mecanismo extensor do joelho e está sujeita a forças biomecânicas intensas durante a contração muscular. Devido a essa ação constante e à força exercida pelo mecanismo extensor, as fraturas que acometem a TAT – especialmente aquelas com desvio ou multifragmentares – apresentam alto risco de falha na fixação, o que pode comprometer a consolidação óssea e a recuperação funcional do paciente. 1 2 3

A literatura atual apresenta divergências quanto ao método ideal para o tratamento dessas fraturas desviadas da TAT. As opções terapêuticas incluem fixação com parafusos de tração, canulados ou não; uso de placas combinadas com parafusos; técnicas de banda de tensão convencionais; ou ainda a reinserção do ligamento patelar à tíbia por meio de âncoras. 4 5 Contudo, não há consenso claro sobre qual dessas abordagens oferece melhor estabilidade mecânica e melhores resultados clínicos, especialmente em fraturas multifragmentares, nas quais a fixação com métodos convencionais de fixação não é possível.

O objetivo desta nota técnica é analisar a eficácia da técnica cirúrgica de fixação com placa de minifragmentos posicionada horizontalmente em banda de tensão na manutenção da redução e na consolidação óssea de fraturas desviadas da TAT em pacientes adultos.

## Materiais e Métodos

Foram incluídos no presente estudo dois pacientes adultos portadores de fratura metafisária proximal da tíbia, caracterizada por desvio e/ou fragmentação da TAT. A seleção dos casos foi realizada de forma retrospectiva, com análise de prontuários médicos, exames clínicos ambulatoriais e exames radiográficos. Foram incluídos pacientes de um hospital terciário, tratados cirurgicamente com a técnica de fixação utilizando a placa de minifragmentos posicionada horizontalmente, de acordo com o princípio da banda de tensão. O acompanhamento dos pacientes foi realizado até a consolidação clínica e radiográfica da fratura (máximo de 1 ano), avaliada com imagens seriadas em radiografias convencionais.

A aprovação ética para publicação do artigo foi obtida sob o CAAE 93952425.0.0000.5125.

### Apresentação dos Casos

#### Paciente 1

O primeiro caso refere-se a um paciente do sexo masculino, 38 anos, vítima de acidente motociclístico, que evoluiu com fratura exposta da região proximal da perna esquerda, classificada como Gustilo-Anderson tipo IIIA. À admissão, observavam-se dois ferimentos cutâneos com exposição óssea na topografia da fratura, sem sinais clínicos de comprometimento neurovascular. O paciente foi submetido, em regime de urgência, a desbridamento de tecidos desvitalizados associado à fixação externa transarticular, com o objetivo de estabilização inicial da lesão e controle local de danos.

Realizou-se um segundo procedimento cirúrgico após 3 dias, com novo desbridamento, retirada do fixador externo e conversão para fixação interna definitiva. A estratégia de fixação combinou duas placas em ponte (medial e lateral) para a fixação do componente metafisário das colunas medial e lateral da tíbia proximal, associadas a placas de minifragmentos com fixação fragmento-específico, além de uma placa horizontal para a fixação da fratura multifragmentar da TAT. Devido ao grau de cominuição, com o intuito de maior estabilidade na fixação, a placa de minifragmentos foi passada dentro do ligamento patelar (transtendão).

**Fig. 1:**
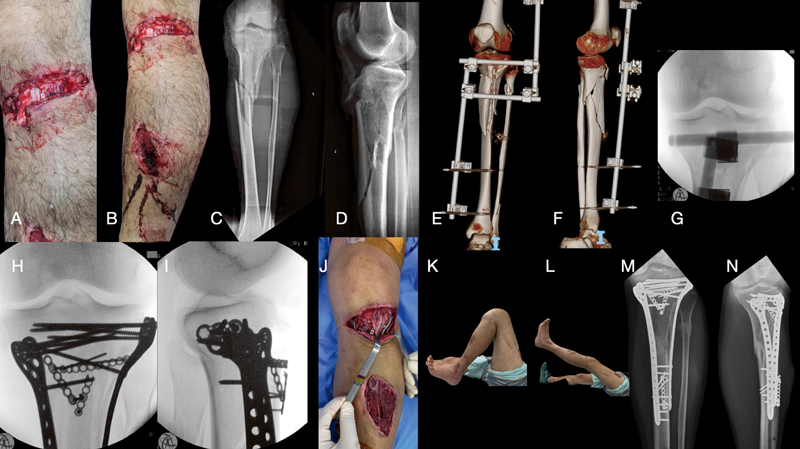
Primeiro caso. ( **A** , **B** ) Fotografias pré-operatórias ilustrando feridas na região anterior da perna esquerda, com exposição da tuberosidade anterior da tíbia (TAT). ( **C** , **D** ) Radiografias da perna esquerda nas incidências anteroposterior (AP) e perfil, evidenciando fratura proximal da tíbia com elevado grau de multifragmentação e extensão diafisária. ( **E** , **F** ) Tomografia computadorizada com reconstrução tridimensional (3D) demonstrando a fragmentação da TAT. ( **G** ) Imagem fluoroscópica do joelho esquerdo na incidência AP após a aplicação de fixador externo transarticular. ( **H** , **I** ) Imagens intraoperatórias mostrando a redução anatômica da superfície articular, com fixação realizada por meio de placas de minifragmentos 2,4 mm Evos Mini Plating System associadas a duas placas 3,5 mm Evos Plating System em ponte nas colunas medial e lateral da tíbia proximal (Smith & Nephew). ( **J** ) Observa-se a placa horizontal posicionada segundo o princípio da banda de tensão, passada por dentro do ligamento patelar, junto à sua inserção na TAT, com o objetivo de conter a fratura multifragmentar. ( **K** , **L** ) Fotografias pós-operatórias evidenciando adequada cicatrização das partes moles e recuperação funcional do joelho, com restabelecimento completo da amplitude de movimento. ( **M** , **N** ) Radiografias nas incidências AP e perfil, demonstrando consolidação completa da fratura, manutenção da redução da TAT e nenhum sinal de falha da fixação.

No pós-operatório, foi instituída terapia por pressão negativa na ferida distal da perna, seguida posteriormente de enxertia cutânea para cobertura definitiva das partes moles. A Fig. 1 ilustra os aspectos do trauma inicial e as etapas do tratamento cirúrgico realizadas no paciente 1.

Com a cicatrização completa da fratura, o paciente foi submetido à retirada da placa em banda de tensão (17 meses após a fixação) com o intuito de minimizar o risco de lesões adicionais por atrito ao ligamento patelar.

#### Paciente 2

**Fig. 2:**
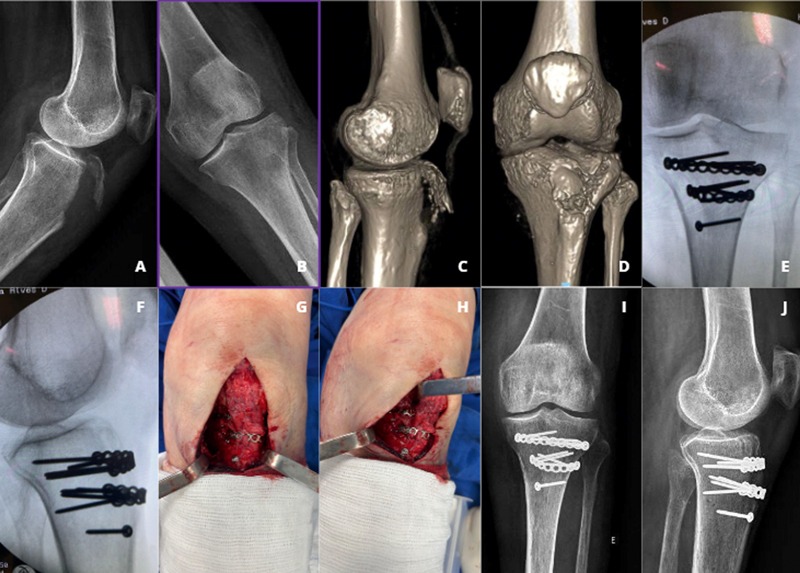
Segundo caso. ( **A** , **B** ) Radiografias do joelho esquerdo nas incidências anteroposterior (AP) e perfil, evidenciando fratura desviada da tuberosidade anterior da tíbia (TAT). ( **C** , **D** ) Imagens de tomografia computadorizada com reconstrução tridimensional, demonstrando o desvio e a multifragmentação da TAT. ( **E** , **F** ) Imagens fluoroscópicas nas incidências anteroposterior e perfil após a fixação da TAT com placa de minifragmentos posicionada horizontalmente, aplicada segundo o princípio da banda de tensão. ( **G** , **H** ) Fotografias intraoperatórias ilustrando o acesso cirúrgico e a disposição final das placas após a fixação. ( **I** , **J** ) Radiografias de controle do joelho esquerdo nas incidências AP e perfil, evidenciando manutenção da redução, ausência de falha do implante e consolidação óssea satisfatória.

O segundo caso refere-se a uma paciente do sexo feminino, 82 anos, portadora de Alzheimer e osteoporose, previamente deambuladora com auxílio de bengala, embora com limitação funcional. A paciente sofreu queda, com trauma direto no joelho esquerdo, evoluindo com desvio e multifragmentação da TAT. Foi realizado tratamento cirúrgico definitivo com redução aberta e fixação interna da TAT, utilizando 2 placas de minifragmentos de 2,4 mm (Evos Mini Plating System *,* Smith & Nephew) posicionadas segundo o princípio da banda de tensão, conferindo adequada estabilidade e manutenção da redução, conforme evidenciado pela avaliação fluoroscópica intraoperatória. Neste caso, não houve necessidade da aplicação transtendão patelar pelas placas horizontais. A Fig. 2 ilustra os aspectos do trauma e as etapas do tratamento cirúrgico realizadas na paciente 2.

Assim como o paciente 1, ambas as placas foram removidas após a consolidação da fratura (3 meses da fixação) para evitar lesão adicional devido ao atrito com o ligamento patelar.

## Discussão

A literatura disponível sobre a avaliação da eficácia e dos resultados em curto e longo prazo dos diferentes métodos de tratamento para fraturas desviadas e/ou multifragmentares da TAT em pacientes adultos é limitada, sendo composta predominantemente por relatos de caso e pequenas séries clínicas, sem estudos comparativos ou evidência de maior nível. 4 5 Essa escassez de dados dificulta a padronização do manejo dessas lesões e reforça a necessidade de investigação de estratégias alternativas que sejam capazes de proporcionar estabilidade adequada, manter a redução e favorecer a consolidação óssea em fraturas biomecanicamente desafiadoras. A utilização de placas posicionadas horizontalmente no tratamento das fraturas proximais da tíbia não representa um conceito novo.

Técnicas que utilizam placas com configuração horizontal e semicircular (em barril, *hoop plate* ou *rim plate* ) vêm sendo propostas para o tratamento de fraturas complexas da região proximal da tíbia. 4 5 6 7 Giordano et al. 6 descreveram o conceito da *hoop plate* como método para conter o perímetro posterior nas fraturas do planalto tibial, atuando como um elemento de contenção periférica dos elementos posteriores. De forma semelhante, Rojas et al. 7 descreveram a *umbrella technique* para fraturas complexas da tíbia proximal, combinando uma placa anterior em configuração tipo *hoop* associada a uma haste intramedular, com o objetivo de aumentar a estabilidade global da construção e conter o perímetro anterior durante a inserção da haste intramedular.

A técnica aqui apresentada obedece a um princípio biomecânico distinto, funcionando como uma verdadeira banda de tensão, que “abraça” a fratura multifragmentar da TAT, promovendo contenção dinâmica dos fragmentos, mesmo na flexão do joelho. O termo *banda de tensão* foi utilizado com base no princípio biomecânico clássico descrito nos fundamentos da AO Foundation, no qual um implante posicionado na zona de tensão de um osso é capaz de converter forças de tração em forças compressivas no foco de fratura durante a mobilização funcional. No caso das fraturas da TAT, durante a flexão do joelho ocorre tração exercida pelo aparelho extensor, gerando forças de tensão na cortical anterior proximal da tíbia. Quando uma placa é posicionada horizontalmente sobre a TAT, ela passa a atuar como um dispositivo de banda de tensão, neutralizando essas forças e promovendo compressão dinâmica no foco de fratura durante a contração do quadríceps e a mobilização do joelho.

Essa configuração pode utilizar uma ou duas placas, a depender do tamanho dos fragmentos e da magnitude necessária da fixação. Além disso, devido ao seu baixo perfil (2,0 ou 2,4 mm), a placa pode, quando necessário, atravessar a substância do tendão patelar, sem comprometer significativamente as partes moles, ampliando as possibilidades técnicas em cenários complexos. Entretanto, não há conhecimento de relatos prévios na literatura da aplicação de placas horizontais segundo o princípio da banda de tensão para a fixação de fraturas da TAT.

O presente estudo apresenta limitações inerentes ao seu desenho. Trata-se de uma nota técnica avaliando a eficácia de um método alternativo de fixação em apenas dois pacientes, sem um grupo comparativo submetido a técnicas convencionais. Além disso, os pacientes não foram avaliados em longo prazo, nem foram aplicados escores funcionais ou instrumentos validados de qualidade de vida. O reduzido tamanho amostral também inviabiliza a realização de análises estatísticas inferenciais, restringindo a interpretação dos resultados a uma análise descritiva. Nos dois casos apresentados, a fixação com placa horizontal em banda de tensão foi capaz de manter a redução, proporcionar estabilidade adequada e permitir a consolidação óssea, sem evidência de falha do implante. Esses achados iniciais sugerem que a técnica pode representar uma opção viável no arsenal terapêutico e reforçam a necessidade de estudos futuros, com maior número de pacientes, seguimento prolongado e avaliações funcionais comparativas, para melhor definir suas indicações e resultados clínicos.

## Considerações Finais

A fixação com placa de minifragmentos posicionadas horizontalmente em banda de tensão demonstrou ser uma alternativa segura e eficaz para o tratamento das fraturas complexas e desviadas da TAT nos dois pacientes avaliados. A técnica proporcionou estabilidade mecânica adequada para a manutenção da redução ao longo do período de seguimento, permitindo a consolidação óssea satisfatória nos casos avaliados.

## References

[1] Schatzker J McBroom R Bruce D The tibial plateau fracture. The Toronto experience 1968–1975 Clin Orthop Relat Res 1979 138 94 104 445923

[2] Ruüdi T P Buckley R Moran C G AO Principles of Fracture Management. 2. ed Davos, Suíça AO Publishing/Thieme 2007

[3] Krettek C Schandelmaier P Nunchuck K Tscherne H Biomechanical evaluation of internal fixation techniques for tibial plateau fractures J Orthop Trauma 1997 11 05 324 330

[4] Kim Y Yoon Y C Cho J W Rim Plate Augmentation of the Posterolateral Bare Area of the Tibial Plateau Using a 3.5-mm Precontoured Locking Compression Plate: A Cadaveric Study J Orthop Trauma 2018 32 05 e157 e160 10.1097/BOT.0000000000001129 29356799

[5] Pires R E Giordano V Santos JKd Labronici P J Andrade MAd Lourenço PRTd Expanding indications of the horizontal belt plate: a technical note Injury 2015 46 10 2059 2063 10.1016/j.injury.2015.06.024 26115580

[6] Giordano V Schatzker J Kfuri M The “Hoop” Plate for Posterior Bicondylar Shear Tibial Plateau Fractures: Description of a New Surgical Technique J Knee Surg 2017 30 06 509 513 10.1055/s-0036-1593366 27685766

[7] Rojas D G Pesantez R Zamorano A The “umbrella” technique: reducing hoop stress during suprapatellar nailing in complex proximal tibial fractures Eur J Orthop Surg Traumatol 2025 35 01 61 10.1007/s00590-025-04180-0 39907802

